# Climate warming suppresses abundant soil fungal taxa and reduces soil carbon efflux in a semi‐arid grassland

**DOI:** 10.1002/mlf2.12098

**Published:** 2023-12-29

**Authors:** Yunpeng Qiu, Kangcheng Zhang, Yunfeng Zhao, Yexin Zhao, Bianbian Wang, Yi Wang, Tangqing He, Xinyu Xu, Tongshuo Bai, Yi Zhang, Shuijin Hu

**Affiliations:** ^1^ College of Resources and Environmental Sciences Nanjing Agricultural University Nanjing China; ^2^ Ningxia Yunwu Mountains Grassland Natural Reserve Administration Guyuan China; ^3^ State Key Laboratory of Loess and Quaternary Geology, Institute of Earth Environment Chinese Academy of Sciences Xi'an China; ^4^ Research Center for Advanced Science and Technology The University of Tokyo Tokyo Japan; ^5^ Department of Entomology & Plant Pathology North Carolina State University Raleigh North Carolina USA

**Keywords:** climate warming, microbial diversity, precipitation reduction, soil carbon dynamics

## Abstract

Soil microorganisms critically affect the ecosystem carbon (C) balance and C‐climate feedback by directly controlling organic C decomposition and indirectly regulating nutrient availability for plant C fixation. However, the effects of climate change drivers such as warming, precipitation change on soil microbial communities, and C dynamics remain poorly understood. Using a long‐term field warming and precipitation manipulation in a semi‐arid grassland on the Loess Plateau and a complementary incubation experiment, here we show that warming and rainfall reduction differentially affect the abundance and composition of bacteria and fungi, and soil C efflux. Warming significantly reduced the abundance of fungi but not bacteria, increasing the relative dominance of bacteria in the soil microbial community. In particular, warming shifted the community composition of abundant fungi in favor of oligotrophic *Capnodiales* and *Hypocreales* over potential saprotroph *Archaeorhizomycetales*. Also, precipitation reduction increased soil total microbial biomass but did not significantly affect the abundance or diversity of bacteria. Furthermore, the community composition of abundant, but not rare, soil fungi was significantly correlated with soil CO_2_ efflux. Our findings suggest that alterations in the fungal community composition, in response to changes in soil C and moisture, dominate the microbial responses to climate change and thus control soil C dynamics in semi‐arid grasslands.

## Impact statement

Semi‐arid grasslands play a critical role in the global carbon (C) cycle and C‐climate feedback. Understanding the responses of soil microorganisms to warming and rainfall change is key to evaluating and predicting soil C dynamics in semi‐arid grasslands under future climate change scenarios. Our study showed that warming induced a shift in the abundant fungal community, favoring oligotrophic fungi (i.e., *Capnodiales* and *Hypocreales*) over the potential saprotrophic *Archaeorhizomycetales*, and reduced C efflux. These findings advance our understanding of soil microbial and C responses to climate change drivers and may help predict and possibly manage soil C sequestration in semi‐arid grasslands.

## INTRODUCTION

Soil microorganisms, mainly bacteria and fungi, dominate litter decomposition and nutrient mineralization in soil^1^, ^2^ and critically affect terrestrial carbon (C) balance and C‐climate feedback^3^, ^4^. Both soil bacteria and fungi are sensitive to changes in soil environments induced by climate change^1^. Yet, they may respond divergently to climate change drivers such as warming and precipitation changes because they have distinct morphologies, cell structures, and physiological traits^1^, ^5^, ^6^. For example, fungi are more resistant than bacteria to water stress induced by warming and reduced precipitation because fungi have thick cell walls and extensive hyphal networks to access nutrients and water over long distances^7^, ^8^. Also, within bacterial taxa, Gram‐positive (G^+^) bacteria have strong, thick, interlinked peptidoglycan cell walls and are more resistant to warm and dry conditions than Gram‐negative (G^−^) bacteria^7^, ^9^. In addition, because fungi have a higher biomass C:N ratio than bacteria, bacteria and fungi have different requirements for C and N^4^. This difference often leads to different C use efficiency between bacteria and fungi, and soil microbial communities with higher fungal‐to‐bacterial biomass ratios are more conducive to soil C retention^3^, ^10^.

Soil bacterial and fungal communities are highly diverse with both abundant and rare taxa coexisting on the surface of or inside soil particles and aggregates^11^, ^12^. However, abundant and rare taxa are not evenly distributed, and the soil microbial community is generally composed of large number of rare species and a few highly abundant species^11^. While microbial taxa with low abundance are often referred to as the rare biosphere and comprise the majority of the Earth's biodiversity, the highly abundant ones are called abundant biosphere^11^, ^13^. Taxa that contribute less than 0.01% or 0.1% to the whole community are often perceived as rare biosphere^11^, ^14^. Both abundant and rare microbial taxa contribute to degrading and mineralizing organic materials and modulate the C‐climate feedback^15^, ^16^, ^17^. Previous studies have largely focused on the abundant members of the soil microbial communities because they often dominate the microbial biomass and presumably dictate biogeochemical C and nutrient cycling^16^, ^18^. Emerging evidence has, however, shown that rare species may play a disproportionately important role^15^, ^17^. Despite its low abundance, rare taxa provide a tremendous reservoir of genetic and functional diversity that allows microbial communities to respond rapidly to environmental change^15^, ^19^, ^20^, and may function as keystone species to maintain ecosystem stability and function^21^, ^22^. For example, Xiong et al.^19^ found that rare species of soil fungi are particularly sensitive to warming in an alpine meadow on the Tibetan Plateau. Similarly, precipitation reduction (Pr) can also alter abundant and rare microbial taxa in soil differentially^8^, ^23^. In a subtropical forest, Zhao et al.^23^ reported that Pr impacted dominant fungal taxa and rare bacterial taxa, but had no effect on rare fungal taxa and abundant bacteria. Meanwhile, rare taxa are likely to be functionally dissimilar from the abundant ones, which may enhance the functionality of the abundant microorganisms^15^, ^24^. Therefore, understanding the differential responses of abundant and rare taxa to climate change drivers may be key to better predicting terrestrial C dynamics and the C‐climate feedback under future climate change scenarios.

Global surface temperature has increased by around 1.09°C since industrialization and is projected to continue to increase by another 1.4–5.8°C by the end of this century^25^. Climate warming is often associated with changes in water balance, that is, by enhancing evapotranspiration and altering precipitation regimes^25^, ^26^. Among the global regions, the most vulnerable to the ongoing climate change are those at the mid‐to‐high latitudes, such as the Loess Plateau in Northwest China^27^, ^28^. The Loess Plateau covers an area of ca. 640,000 km^2^ and its deep soil largely originates from the accumulation of wind‐blown dust^29^. In addition to long‐term human disturbance, low rainfall with extreme uneven distribution limits plant growth, leading to the most serious soil erosion in the world^30^. Over the last five decades, the average temperature on the Loess Plateau has increased by ca. 1.9°C^27^, as compared to the increase of 1.09°C in the average global temperature. Concurrent with climate warming, this region has also experienced more extreme rainfall patterns with extensive periods of drought followed by heavy precipitation events^28^. Climate warming and precipitation change may alter soil physical and chemical properties (i.e., water content and nutrient substrate), plant growth, and plant‐derived C input to soil^31^, ^32^. Alterations in soil resources, particularly organic C and soil moisture, can profoundly influence the growth and activities of soil microorganisms, and their interactions with the environment, which largely control the fate of recent plant‐derived C inputs and also affect the turnover of the soil organic C protected in the soil matrix^1^, ^33^, ^34^.

Although many field studies have examined the impact of climate change drivers, such as warming and rainfall change, on plant productivity and community composition^35^, ^36^, the response of soil microorganisms, the subcommunities of abundant and rare microbes in particular, have received limited attention^20^. We initiated a field experiment in a semi‐arid grassland on Loess Plateau to examine the impact of warming, Pr, and their combination on the plant, bacterial, and fungal communities. Our objectives were to 1) determine the direct effects of warming and Pr on soil microbial biomass, activities, and communities of abundant and rare taxa; and 2) examine the potential linkages among plant growth, soil microbial communities, particularly abundant and rare microbial taxa, and soil C dynamics under warming and Pr. We hypothesized that 1) both warming and Pr are more suppressive to bacteria than fungi, increasing fungal dominance but reducing total microbial CO_2_ efflux; 2) warming and Pr alter the relative composition of abundant and rare bacteria and fungi in favor of rare taxa; and 3) alterations in the microbial community composition correlate with soil C efflux.

## RESULTS

### Responses of soil chemical, microbial properties, and C efflux to warming and Pr

Across all the four treatments, NO_3_^−^‐N (11.1 mg N kg^−1^ soil) was more dominant than NH_4_^+^‐N (2.41 mg N kg^−1^ soil) (Table 1). Warming, but not Pr, significantly reduced soil NH_4_^+^‐N by 16.2% (Table 1). Warming alone tended to increase soil NO_3_^−^‐N by 16.7% and Pr significantly enhanced soil NO_3_^−^‐N (Table 1). While Pr had no effect on soil dissolved organic C (DOC), net mineralization rate (NMR), or microbial biomass C (MBC), warming significantly enhanced soil DOC but reduced MBC and NMR (all *p* < 0.05; Table 1). Also, warming significantly reduced the MBC:MBN ratio (*p* < 0.05; Table 1), suggesting a decrease in the relative dominance of fungi over bacteria. In contrast, Pr significantly reduced MBN, leading to a marginal increase in the ratio of MBC to MBN (*p* = 0.06; Table 1).

**Table 1:** Effects of warming and precipitation reduction on soil properties.

	Treatments				Two‐way ANOVA			
Variable	CK	Pr	W	WPr	Warming	Pr		Warming × Pr
NH_4_^+^‐N (mg N kg^−1^)	2.57 ± 0.24	2.67 ± 0.26	2.49 ± 0.03	1.90 ± 0.08	**0.04**	0.21		*0.08*
NO_3_^−^‐N (mg N kg^−1^)	9.0 ± 0.5	12.9 ± 1.4	10.5 ± 0.6	12.1 ± 1.1	0.72	**0.01**		0.26
DOC (mg C kg^−1^)	140.4 ± 3.4	151.8 ± 20.2	173.4 ± 9.5	176.7 ± 10.5	**0.04**	0.57		0.75
NMR (mg N kg^−1^ day^−1^)	1.19 ± 0.14	1.18 ± 0.22	0.84 ± 0.04	0.90 ± 0.06	**0.04**	0.85		0.80
MBC (mg C kg^−1^)	698.2 ± 67.6	803.5 ± 40.0	584.0 ± 79.7	511.2 ± 89.8	**0.02**	0.83		0.24
MBN (mg N kg^−1^)	160.4 ± 20.9	118.2 ± 3.9	159.1 ± 8.2	125.5 ± 14.4	0.83	**0.02**		0.75
MBC/MBN	4.7 ± 0.9	6.8 ± 0.2	3.7 ± 0.5	4.0 ± 0.6	**0.01**	*0.06*		0.16

Data presented as mean values with standard errors (*n* = 4). Significant effects (*p* < 0.05) are shown in bold, and marginally significant effects (0.05 < *p* ≤ 0.10) are in italics. ANOVA, analysis of variance; C, carbon; CK, control; DOC, dissolved organic carbon; MBC, microbial biomass carbon; MBN, microbial biomass nitrogen; N, nitrogen; NMR, net nitrogen mineralization rate; CK, control; Pr, precipitation reduction; W, warming; WPr, a combination of warming and precipitation reduction.

**Figure 1:**
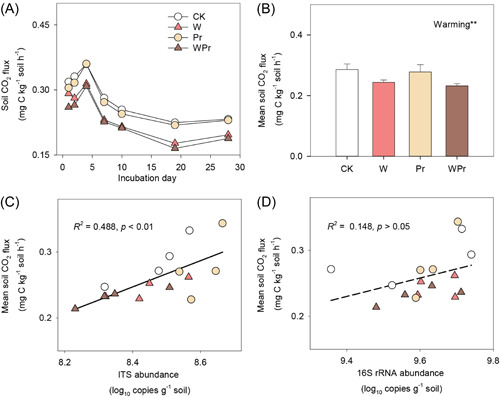
Soil CO_2_ flux and the relationships between soil CO_2_ flux and fungal or bacterial abundance. Effects of warming and Pr on soil CO_2_ flux (A, B), and the relationships between mean soil CO_2_ flux and soil fungal (C) or bacterial abundance (D), respectively are shown. Error bars in (B) show standard errors of the means (*n* = 4). The statistically significant effects of warming, Pr, and their interaction (warming × Pr) are indicated: **0.001 < *p* ≤ 0.01. ITS, internal transcribed spacer.

We measured soil C efflux using soil samples collected after 5‐year field treatments and through an incubation experiment. Soil CO_2_ fluxes varied from 0.165 to 0.365 mg C kg^−1^ soil h^−1^ during the 28‐day incubation period and peaked in all the treatments at Day 4 (Figure 1A). On average, warming significantly reduced soil CO_2_ emission by 15.6%, but Pr had no effect on it (Figure 1B). There was no significant interaction between warming and Pr on soil CO_2_ emission (Figure 1B).

### Responses of soil bacterial and fungal abundances to warming and Pr

**Figure 2:**
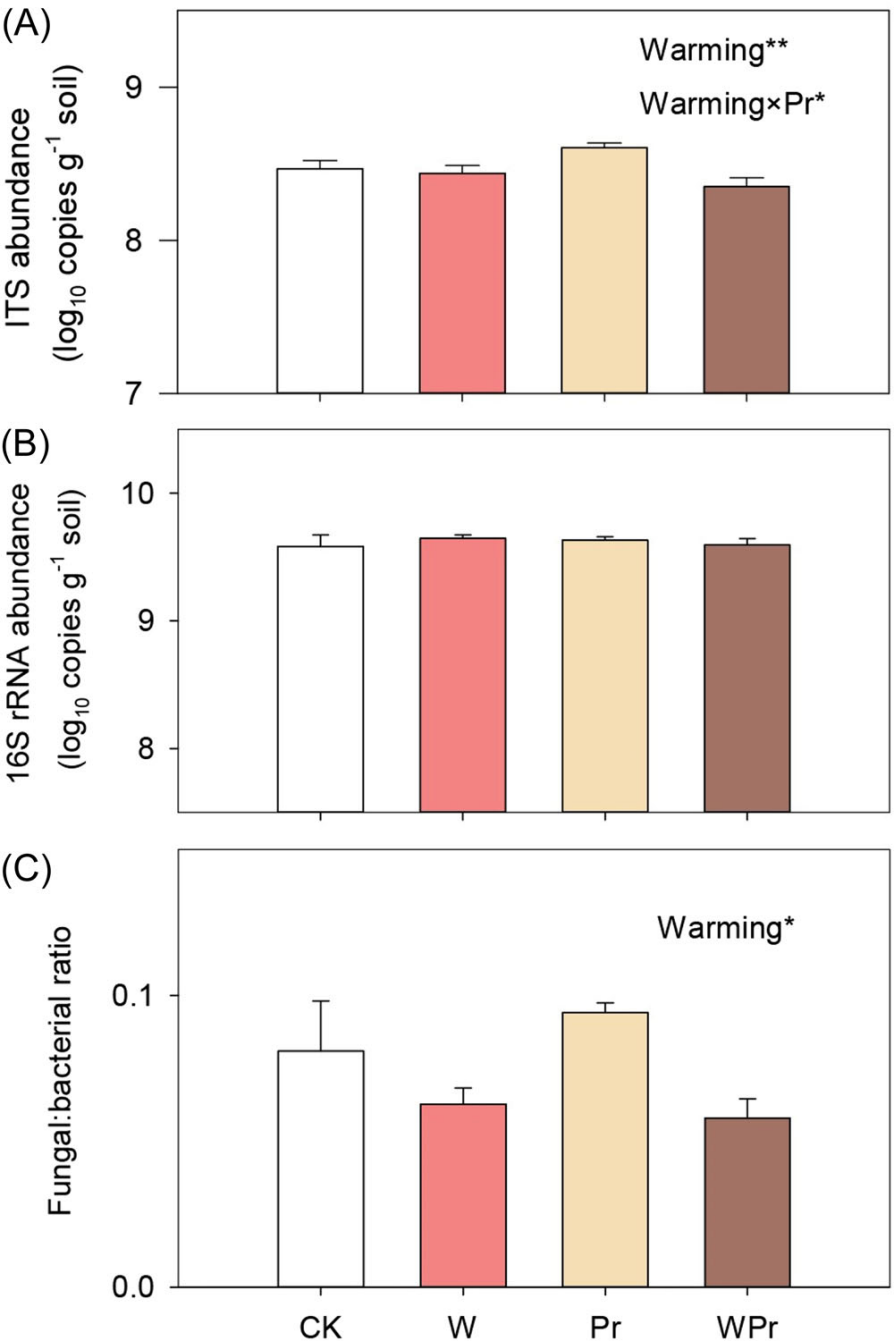
Soil fungal and bacterial abundance, and the ratio of fungal to bacterial abundance under different treatments. Effects of warming and precipitation reduction on the abundance of soil fungi (A), bacteria (B), and the ratio of fungi to bacteria (C) are shown. Error bars show standard errors of the means (*n* = 4). The statistically significant effects of warming, Pr, and their interaction (warming × Pr) are indicated: *0.01 < *p* ≤ 0.05; **0.001 < *p* ≤ 0.01.

Neither warming nor Pr significantly affected soil bacterial abundance (Figure 2B and Table S2). Warming reduced soil fungal abundance by 27.6% (*p* < 0.05; Figure 2A and Table S2). There was an interaction between warming and Pr on soil fungal abundance (*p* < 0.05; Figure 2A and Table S2). In addition, warming significantly decreased the ratio of fungi to bacteria by 31.1%, but Pr had no effect on it (Figure 2C and Table S2).

### Responses of abundant and rare soil microbial taxa to warming and Pr

**Figure 3:**
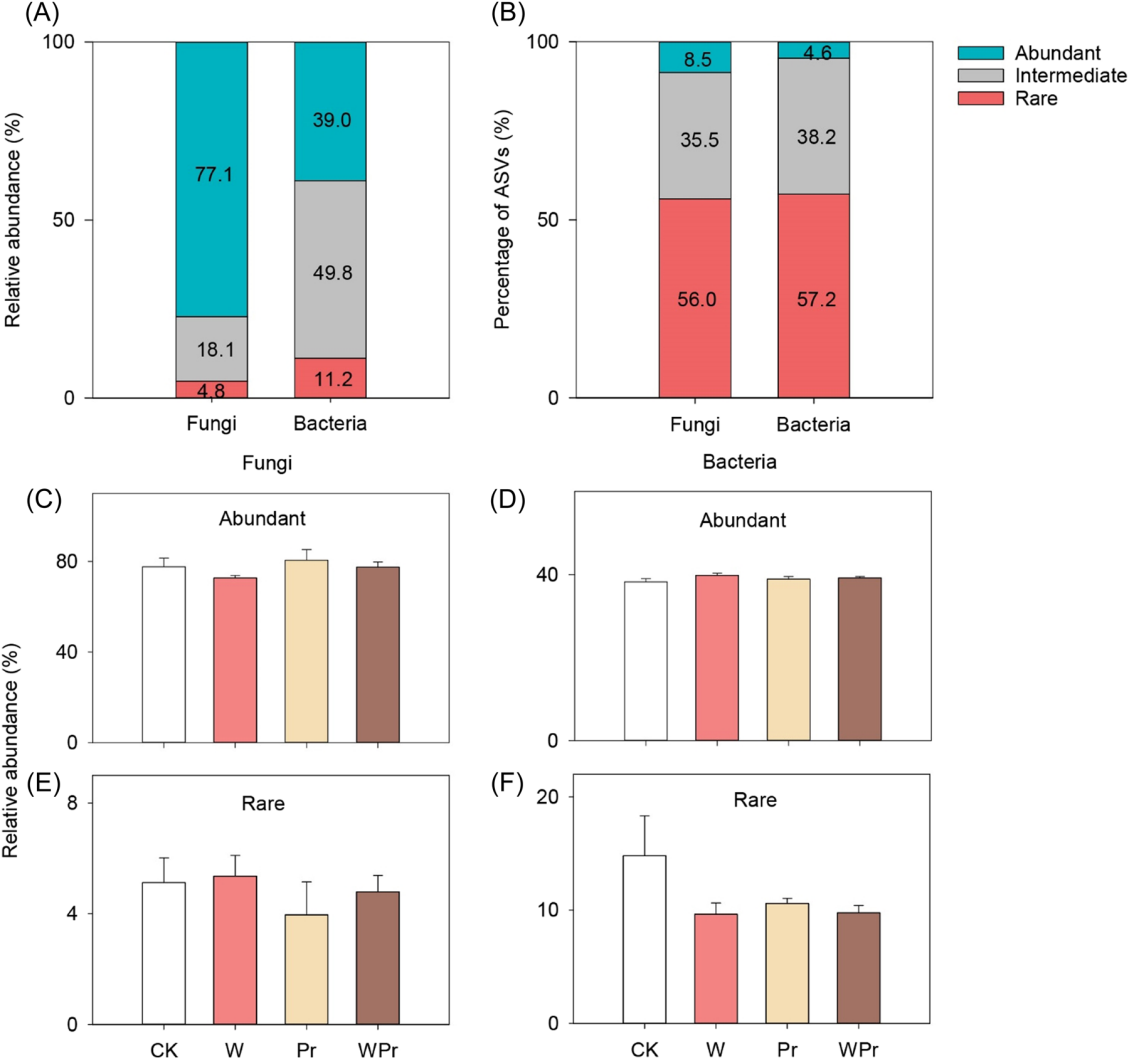
Relative abundance of abundant and rare taxa of soil fungi and bacteria. (A, B) The relative abundance and amplicon sequence variants (ASVs) percentages of abundant and rare taxa across the four treatments. (C–F) Effects of warming and Pr on the relative abundance of abundant and rare taxa of soil fungi and bacteria. Error bars show standard errors of the means (*n* = 4).

For soil fungi, abundant taxa accounted for 77.1% of the sequence reads with a significantly lower proportion of amplicon sequence variants (ASVs) (8.5%), and rare taxa comprised 4.8% of the sequence reads with a significantly higher proportion of ASVs (56.0%) (Figures 3A,B). In comparison, abundant bacterial taxa accounted for 39.0% of the sequence reads with a significantly lower proportion of ASVs (4.6%), and rare bacterial taxa comprised 11.2% of the sequence reads with a significantly higher proportion of ASVs (57.2%) (Figures 3A,B).

Warming tended to reduce the abundance of abundant fungi by 5.1% (Figure 3C), and Pr decreased the abundance of rare fungi by 16.6% (Figure 3E). Neither warming nor Pr significantly impacted the abundance of abundant bacteria (Figure 3D). Warming and Pr tended to decrease the relative abundance of rare bacteria by 23.5% and 16.8%, respectively (Figure 3F).

### Responses of alpha diversities of abundant and rare soil fungi and bacteria to warming and Pr

Across all the four treatments, rare communities of fungi and bacteria had higher richness and Shannon indexes than abundant communities of fungi and bacteria, respectively (Figures S2 and S3). Neither warming nor Pr significantly influenced the alpha diversity indices of abundant or rare fungi (i.e., richness, Shannon, and Simpson indexes) (Figures S2 and Table S4). While warming and Pr had no effect on alpha diversities of rare bacteria, warming significantly increased the Simpson index and marginally increased the Shannon index of abundant bacteria (Figure S3 and Table S5).

### Responses of beta diversities of abundant and rare soil fungi and bacteria to warming and Pr

**Figure 4:**
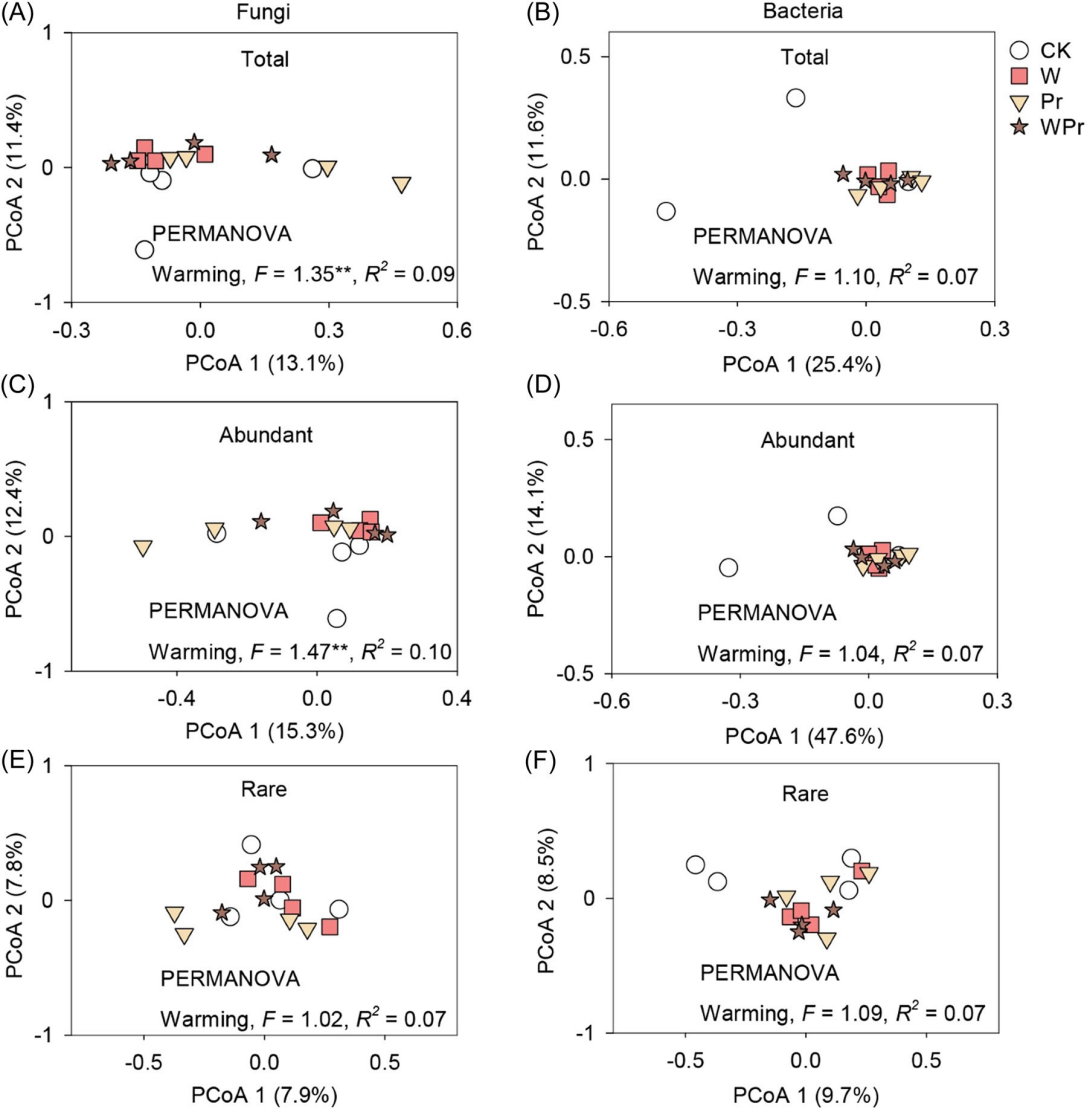
Soil fungal and bacterial community composition. Effects of warming and Pr on soil fungal and bacterial community composition (A, B: total; C, D: abundant; E, F: rare) are shown. The community composition of soil fungi and bacteria was assessed by PCoA at the taxon level based on the Bray–Curtis dissimilarity. The effects of warming and Pr on microbial composition were determined using PERMANOVA (*F*, *R* ^2^, and *p* values; **p < 0.01). PERMANOVA, permutational analyses of the variance.

Warming and Pr differently affected the beta diversities of fungi and bacteria. Permutational analyses of the variance (PERMANOVA) showed that warming significantly altered the abundant but not rare community composition of soil fungi, while Pr affected neither of them (Figure 4A,C,E). PCoA revealed that the community composition of soil‐abundant fungi was noticeably separated on axes 1 and 2, with 15.3% and 12.4% interpretations on axes 1 and 2, respectively (Figure 4C). In addition, neither warming nor Pr had significant effects on the community composition of abundant or rare soil bacteria (Figure 4B,D,F).

**Figure 5:**
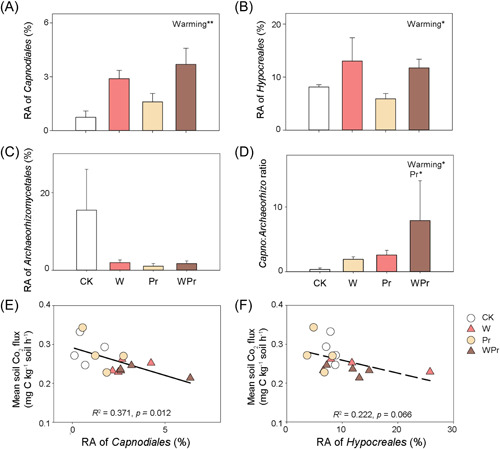
Relative abundance (RA) of abundant fungal *Capnodiales*, *Hypocreales*, *Archaeorhizomycetales*, and the ratio of *Capnodiales* to *Archaeorhizomycetales*. Effects of warming and Pr on the RA of *Capnodiales* (A), *Hypocreales* (B), *Archaeorhizomycetales* (C), and the ratio of *Capnodiales* to *Archaeorhizomycetales* (D) in abundant soil fungal community, and the relationships between soil CO_2_ efflux and the RA of *Capnodiales* (E) and *Hypocreales* (F), respectively. Error bars show standard errors of the means (*n* = 4). The statistically significant effects of warming, Pr, and their interaction (warming × Pr) are indicated: *0.01 < *p* ≤ 0.05; **0.001 < *p* ≤ 0.01.

In the fungal communities, *Ascomycota* (62.5%), *Mortierellomycota* (16.5%), and *Basidiomycota* (10.6%) were the most prevalent abundant fungal phyla (Figure S4). The dominant rare fungal taxa were *Ascomycota* (46.1%), *Basidiomycota* (15.9%), *Mortierellomycota* (1.7%), and *Chytridiomycota* (1.5%) (Figure S4). At the fungal order level, warming significantly increased the relative abundance of abundant *Capnodiales* and *Hypocreales*, but tended to decrease that of *Archaeorhizomycetales* (Figure 5A–C and Table S6). Warming and Pr significantly increased the ratio of *Capnodiales* to *Archaeorhizomycetales* (Figure 5D). Additionally, warming significantly increased the relative abundance of rare *Cantharellales* but decreased rare *Chaetothyriales* (Figures S5 and Table S6).

As for the bacterial communities, the rare community was dominated by phyla *Actinobacteria* (38.9%), *Proteobacteria* (23.2%), *Acidobacteria* (16.6%), and *Chloroflexi* (6.9%), similar to the abundant community with the four phyla accounting for 95.8% of the total sequences (Figure S6). Neither warming nor Pr had any significant impacts on abundant or rare bacterial community composition at the phylum level (Figures S6 and S7).

### Relationships among soil properties, fungal and bacterial abundance and community composition, and CO_2_ efflux

**Figure 6:**
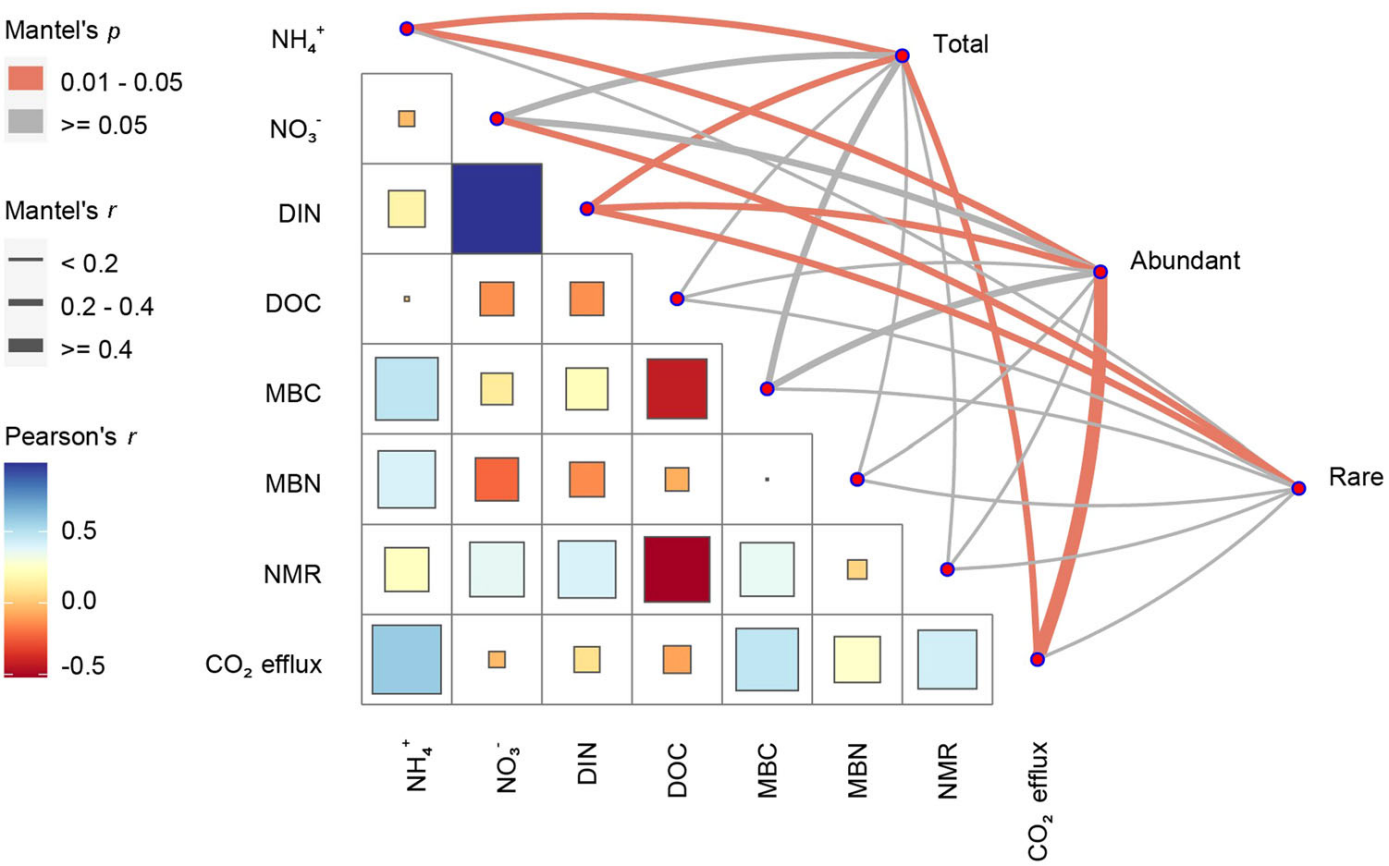
Potential drivers of soil fungal community composition. Relationships between soil fungal community composition (total, abundant, and rare) and soil properties or soil CO_2_ efflux. Edge width corresponds to Mantel's *r* value, and the edge color denotes the statistical significance. Pairwise correlations of these variables are shown with a color gradient presenting Pearson's correlation coefficient. Soil variables include soil ammonium (NH_4_^+^), nitrate (NO_3_^−^), dissolved inorganic N (DIN), dissolved organic C (DOC), microbial biomass C (MBC) and microbial biomass N (MBN), and net mineralization rate (NMR).

The Pearson's correlation analysis showed that soil CO_2_ efflux was significantly and positively correlated with soil available NH_4_^+^‐N and the abundance of soil fungi (Figure 1C,D), but negatively correlated with the relative abundance of soil‐abundant *Capnodiales* (*p* = 0.012) and *Hypocreales* (*p* = 0.066) (Figure 5E,F). Mantel test revealed that abundant but not rare fungal community composition was correlated with soil CO_2_ efflux (Figure 6 and Table S8). Also, the abundant fungal community composition was significantly correlated with soil available NH_4_^+^‐N (Figure 6 and Table S8). However, the rare fungal community composition showed a strong correlation with soil available NO_3_^−^‐N (Figure 6 and Table S8). Furthermore, structural equation model (SEM) analysis showed that the warming‐induced decline in soil CO_2_ efflux was mainly explained by soil moisture, labile C, and the composition of abundant fungal community but not by total fungal abundance (Figure S8).

## DISCUSSION

Microbial responses to climate warming and Pr may have the potential to significantly affect grassland ecosystem productivity and C dynamics. So far, studies have mainly focused on quantifying the alterations of soil microbial processes (e.g., soil respiration and N‐cycling processes)^37^, ^38^. Yet, the linkages among plant growth, soil microbes, and soil C efflux, as influenced by climate warming and Pr, have not been well established.

Results from our field experiment showed that warming suppressed fungi, but not bacteria, and thus reduced soil C efflux in the semi‐arid grassland (Figures 1 and 2). These results were surprising and in contrast with our hypothesis because warming is expected to favor the growth of fungi over bacteria. Multiple mechanisms may have contributed to the observed shift in the microbial community structure (Figures 2 and 4). Warming significantly increased root biomass at our site, while reducing soil moisture across the growing season^39^. This has likely increased root‐derived organic C inputs to the soil as root exudates and fine root turnover increase, as evidenced by higher soil soluble C (Table 1). As bacteria and fungi are associated with different life strategies with bacteria growing and dividing faster than fungi, bacteria may outcompete fungi for more labile C utilization^40^, ^41^ and/or via the production of antibiotics for resources in the rhizosphere^42^, ^43^. Also, evidence is mounting that antibiotics may be the primary driver shaping the soil microbial composition with the potential to affect ecosystem function^7^, ^44^, ^45^. Increased soil labile C availability and reduced moisture may prompt competition among bacteria and fungi for water, and bacterial production of antibiotics may jointly play a major role in shaping the soil microbial community composition^12^, ^44^. Further, according to the Preferential Substrate Utilization Hypothesis^46^, higher labile C and lower soil moisture under warming may promote microbes to utilize available labile C rather than to produce hydrolytic enzymes to decompose complex organic compounds, which may also contribute to the reduced CO_2_ efflux in our study.

Contrary to warming, Pr significantly increased soil MBC (Table 1) but had no effect on abundance, community composition of soil fungi and/or bacteria, or soil C efflux (Figures 1, 2, and 4). These results also contradict our expectation that reduced precipitation favors the growth of soil fungi over bacteria and suppresses CO_2_ efflux (Hypothesis 1). These null effects of Pr on the microbial community indices may be due to a shift in plant community composition in favor of grasses over forbs under Pr at our site^38^. Compared with forbs, most grasses have highly branched fibrous roots, which are more fragile and turn over faster than taproots^38^. High root turnover likely increased plant C allocation belowground, contributing to the observed increase in soil MBC (Table 1). However, grasses have high efficiency in N uptake and may have induced relative N deficiency in microbes, as suggested by the higher MBC:MBN ratio and soil available NO_3_^−^‐N (Table 1). Low N may further limit microbial production of enzymes and decomposition^4^, ^7^, offsetting the positive effect on MBC. In addition, our experiment sits on a typical semi‐arid grassland, where low soil moisture promotes nitrification and induces a dominance of NO_3_^−^‐N over NH_4_^+^‐N (Table 1). Together, our results suggest that while both warming and Pr reduce soil moisture and constrain microbes, their indirect effects through affecting plant C allocation belowground and microbial interactions may play a more significant role in shaping the microbial community.

More interestingly, warming significantly altered the abundant fungal community composition (Figure 4), which significantly correlated with soil CO_2_ emissions (by Mantel test, *p* < 0.05; Figure 6). At this site, Bai et al.^39^ have previously observed that warming‐driven reduction in total fungal biomass (estimated by the phospholipid fatty acids) correlated with decreased soil respiration. However, the relative effects on abundant and rare microbial taxa were not assessed. Different abundant and rare microbial taxa often vary in their sensitivity and strategies to soil temperature and moisture fluctuations^15^, ^20^. Climate warming but not Pr resulted in the preferable growth of order *Capnodiales* and *Hypocreales* over *Archaeorhizomycetales* (Figure 5). *Capnodiales* (*Dothideomycetes*) and *Hypocreales* (*Sordariomycetes*) are considered as the potent degraders of recalcitrant C and important components of soil oligotrophic fungal taxa^47^, ^48^. In a Tibetan alpine meadow, Che et al.^48^ reported that warming increased the proportion of an oligotrophic fungal class *Dothideomycetes* and reduced the proportion of active saprotrophic fungi. Compared with *Capnodiales* and *Hypocreales*, little is known about the ecology of the *Archaeorhizomycetales*, but it has been reported that some *Archaeorhizomycetales* may function as an important potential saprotroph in soil^49^. A decrease in the relative abundance of *Archaeorhizomycetales* under warming may partially contribute to the reduced soil CO_2_ emission (Figures 1B and 5C). Additionally, it has been reported that *Capnodiales* are strong assimilators of plant residue C and may contribute positively to soil organic C sequestration^50^, which may contribute to the negative correlation between soil CO_2_ efflux and the relative abundance of *Capnodiales* or *Hypocreales* observed in this study (Figure 5E,F). Moreover, our SEM analysis confirmed that the warming‐induced decline in soil CO_2_ efflux was mainly due to alterations in soil C availability and moisture and abundant fungal community composition (Figure S8).

In summary, our study represents one of the few field experiments that explicitly manipulated air temperature and reduced precipitation to examine the responses of different soil microbial subcommunities and CO_2_ efflux in semi‐arid grasslands. Results from our field study illustrate that warming altered soil microbial communities, favoring the growth of bacteria over fungi. Also, warming induced a shift in the soil‐abundant fungal community composition in favor of *Capnodiales* and *Hypocreales* over potential saprotrophic *Archaeorhizomycetales*, which likely contributed to the reduced soil C efflux*.* Together, these results suggest that changes in soil C availability and moisture may dominate the microbial responses to climate change and thus control soil C dynamics in semi‐arid grasslands under future climate change scenarios.

## MATERIALS AND METHODS

### The study site and the field experimental design

Our field study was carried out in a semi‐arid grassland of Loess Plateau at the Yunwu Mountains Natural Preserve (36°13′ to 36°19′ N, 106°24′ to 106°28′ E; ca. 2000 m a.s.l.), northeast of Guyuan City, Ningxia Hui Autonomous Region of China. This experimental site has a typical semi‐arid climate with a mean annual rainfall of about 455 mm. Approximately 70% of rainfall is concentrated in the growing season from July to September. The mean annual air temperature is 7°C (ranging from −6.5°C in January to 22.8°C in July). The dominant plant species in the semi‐arid grassland are *Artemisia sacrorum, Stipa przewalskyi, Stipa grandis, Chrysanthemus lavandulifolium*, and *Saussurea alata* ^51^. The study area has been fenced to exclude large animal grazing and protected since 1982. The soil at the study site is classified as a montane gray‐cinnamon, Calci‐Orthic Aridisol, or Haplic Calcisol based on the Chinese and FAO classification systems. The soil contained 40.2 g total C kg^−1^, 4.0 g total N kg^−1^, and had a pH of ca. 8.0 (measured in H_2_O). The field experiment was set up on a largely flat hilltop (Figure S1) in early June 2015 as a factorial experiment in a randomized block design with four blocks (replicates)^38^, ^39^, ^52^. Each block contains 12 treatment plots of 4 m × 4 m, separated by a buffer strip 1.5 m wide. There was also a 5 m buffer zone between each block. All treatments were randomly assigned to plots within blocks and consisted of factorial combinations of two temperatures (ambient temperature and elevated temperature), three precipitation levels (precipitation addition [+30%], ambient precipitation, and Pr [−30%]), and two N inputs (control and 12.0 N m^−2^ year^−1^). Each warming treatment was achieved via open‐top chambers (OTCs) because electricity is inaccessible in this very remote field site. A large number of field studies have shown that this type of warming method was effective^39^, ^52^, ^53^, ^54^. The OTCs were made of 6‐mm‐thick transparent polycarbonate materials with high light transmittance (>90%) and no infrared transmittance (<5%). They were hexagonal and had a height of 51.76 cm with a 60 cm width at the top and 75 cm at the bottom (Figure S1). There are four OTCs placed in each plot to ensure sufficient warming (Figure S1). For the Pr treatment, we placed rainout shelters made of seven tilted v‐shaped transparent plexiglass 1 m above the soil surface on an iron hanger over each field Pr plot (see Figure S1 for detail). Our previous publication showed that the rainout shelters intercepted approximately 30% of rainfall considering the wind effects on raindrops and possible lateral transport of water in the field^38^. The rainwater was collected from each Pr plot using plastic containers and manually added to the nearest precipitation addition plot within 24–48 h after the rainfall event ended. Details of field N treatment have been described in previous publications^39^, ^55^. For this study, only four treatments were selected for sampling: (a) CK; (b) W; (c) Pr; and (d) WPr. The Preserve was not accessible during the spring and early summer of 2020. Therefore, air temperature, soil temperature and moisture, and field CO_2_ fluxes were not monitored. However, our previous record showed that warming increased air temperature by 2.0, 2.6, and 2.1°C, and reduced average soil volumetric moisture by 15.1%, 16.8%, and 13.5% in 2018, 2019, and 2021, respectively (Qiu et al. unpublished). It did not consistently increase soil temperature at 10 cm depth in either 2018 or 2019 but significantly increased it by 0.5°C in 2021 (Qiu et al. unpublished). Also, our previous publication^38^ showed that Pr reduced soil moisture by 9.8%, 10.9%, and 7.6% in 2019, 2021, and 2022, respectively.

### Plant and soil sampling

In mid‐August 2020 at the peak of the growing season, soil samples were collected from the field plots. In each plot, three 5‐cm diameter soil cores (0–10 cm depth) were collected, and well mixed to generate a composite sample per plot. All the composite samples were immediately kept in coolers with ice bags and transported to the laboratory for processing. All samples were sieved to pass through a 2 mm sieve, and any visible stones and plant residues were carefully removed from the sieved soil. A small subsample (approximately 50 g) of soil was immediately frozen at −20°C for molecular analysis. The remaining samples were then stored at 4°C and used for the chemical and microbial analyses, which were conducted or initialized within 10 days.

### Soil physical, chemical, and microbial analyses

Soil inorganic‐N (NH_4_^+^‐N and NO_3_^−^‐N) and dissolved organic C (DOC) were determined from 20 g soils (dry soil equivalent), and extracted with 50 ml of K_2_SO_4_ (0.5 M) after 30 min of rotary shaking. Soluble inorganic N and organic C in the extracts were quantified by a flow injection auto‐analyzer (SEAL‐AA3) and a TOC analyzer (Elementar Vario TOC Cube), respectively. Soil microbial biomass C (MBC) and N (MBN) were determined using the chloroform fumigation extraction method^56^. Soil net N mineralization was assessed by the accumulation of inorganic N (NH_4_^+^‐N and NO_3_^−^‐N) during a 4‐week incubation (25°C in the dark) with 20.0 g fresh soil following the method in Zhang et al.^38^

### Soil CO_2_ efflux

As field CO_2_ efflux in 2020 was not determined, in this study, soil CO_2_ efflux rate was measured by the aerobic incubation method^57^. In brief, for each soil sample, an equivalent of 20.0 g dried soil was weighed into a 120‐ml mason jar and adjusted to a moisture level of 60% water‐holding capacity. All jars were then incubated at room temperature of 25°C in the dark for 4 weeks. Soil CO_2_ emissions were measured after 1, 2, 4, 7, 10, 19, and 28 days of incubation following a static procedure as described in Zhang et al.^38^. CO_2_ concentrations were determined on a gas chromatograph (GC; 7890B; Agilent Technologies) equipped with a flame ionization detector.

### Determination of soil fungal and bacterial abundance

For each soil sample, total genomic DNA was extracted from 0.25 g (dry weight) frozen soil using the MoBio Power Soil DNA kit (MoBio Laboratories Inc.) following the manufacturer's instructions. Soil DNA quality and size were checked by electrophoresis on a 1% agarose gel. The abundance of total fungi and bacteria was determined using real‐time quantitative PCR (qPCR) (7300 Real‐Time PCR System; Applied Biosystems) to amplify the fungal internal transcribed spacer (ITS) and bacterial 16S rRNA genes, respectively. Primer sets and PCR conditions for each gene are provided in Table S1. The PCR reactions were performed in triplicate and consisted of 12.5 µl of SYBRs Premix Ex Taq™ (Takara), 0.5 µl of Rox Reference Dye, 10.0 µl of ddH_2_O, 0.5 µl of each primer, and 1 µl of the template DNA, giving a final volume of 25 µl. Standard curves for qPCR were made following the method in Zhang et al.^38^. All PCR reaction efficiencies ranged between 95% and 110% with *R* ^2^ > 0.98.

### Illumina MiSeq sequencing and analyses of fungal and bacterial communities

To characterize soil fungal and bacterial communities through high‐throughput sequencing analysis, the fungal ITS and bacterial 16S rRNA genes were amplified using the same primer sets as for qPCR (see Table S1). All PCR reactions were carried out in triplicate with a total volume of 25 μl containing 5 μl of reaction buffer (5×), 5 μl of GC buffer (5×), 0.25 μl of Q5 DNA polymerase, 2 μl of dNTPs (2.5 mM), 8.75 μl of ddH_2_O, 1 μl (10 μM) of each primer, and 2 μl of template DNA. The PCR amplification conditions were run with a 5 min initial denaturation at 98°C, 25 cycles of denaturation at 98°C for 30 s, annealing at 53°C for 30 s, and extension at 72°C for 45 s, and a final 5 min extension at 72°C. The triplicate PCR products for each sample were combined and purified with Vazyme VAHTSTM DNA Clean Beads (Vazyme). The purified PCR products were then pooled in equimolar and sent for paired‐end sequencing on the Illumina NovaSeq platform with NovaSeq. 6000 SP Reagent Kit (Illumina) at Shanghai Personal Biotechnology Co., Ltd.

Processing of all raw sequencing data was performed with the Quantitative Insights Into Microbial Ecology 2 (QIIME2) version 2019.4.0^58^. In brief, raw sequences were demultiplexed using the demux plugin according to the unique barcodes for each sample. Then, the primers were cut from the sequences by the CUTADAPT plugin^59^. Sequences were quality‐filtered, denoised, merged, and chimera‐filtered using the plugin DADA2^60^. Then, sequences below the quality score of 25 and fewer than 200 bp in length or sequences with ambiguous nucleotides were removed. After denoising, the filtered sequences were clustered into ASVs. The UNITE (http://unite.ut.ee/index.php)^61^ and SILVA databases (https://www.arb-silva.de/)^62^ were used for fungal and bacterial taxonomy assignment.

The fungal and bacterial ASV tables were rarefied to 51,171 and 48,367 per sample for subsequent analyses, respectively. The abundant and rare ASVs were classified into six categories following recent publications^63^, ^64^, ^65^. Briefly, ASVs with relative abundances below 0.01% of the total sequences in all samples were designated as “always rare taxa, ART” and those with relative abundances <0.01% in some samples but never ≥1% in any sample were “conditionally rare taxa, CRT”. ART and CRT were collectively referred to as rare taxa. ASVs with relative abundances ≥0.1% in all samples were designated as “always abundant taxa, AAT” and those with relative abundances ≥0.01% in all samples and ≥0.1% in some samples were “conditionally abundant taxa, CAT”. AAT and CAT were collectively referred to as abundant taxa. The remaining ASVs (0.01%–0.1%) were defined as “intermediate taxa”. In this study, we mainly focused on the abundant and rare taxa.

### Statistical analysis

Data were subjected to analysis of variance (ANOVA) using the stats package in R (version 4.1.2, R Development Core Team)^66^. Data were first tested for homogeneity of variances and normal distribution. Some variables were transformed for normal distribution where necessary. Soil parameters were analyzed using a linear mixed‐effects model with warming and Pr treatments as the fixed factor and block as a random effect. For all tests, the statistical difference was considered significant when *p* < 0.05.

We analyzed changes in the composition and structure of fungal and bacterial communities between treatments by multivariate PERMANOVA (9999 permutations) using the “adonis” function in the vegan package of R^67^. To visualize the differences in soil fungal and bacterial communities between experimental treatments, the principal coordinate analysis (PCoA) using Bray–Curtis distances was employed. For this, we used the “metaMDS” function in the vegan package^67^. Relationships between soil fungal communities and soil variables were examined by the Mantel test with “mantel” function in the vegan package^67^.

SEM analysis was further conducted to examine the effects of warming on soil CO_2_ emission via biotic and abiotic variables. We used PCoA axis 1 scores as a proxy for abundant fungal community composition. The data were fitted to the models using the maximum likelihood estimation method and fitted with the *χ* ^2^ test. The SEM analysis was performed with the lavaan package^68^ in R.

## AUTHOR CONTRIBUTIONS

**Yunpeng Qiu**: Conceptualization (equal); data curation (lead); formal analysis (lead); funding acquisition (lead); investigation (lead); methodology (equal); project administration (lead); resources (equal); software (equal); supervision (equal); validation (equal); visualization (equal); writing—original draft (lead); writing—review and editing (lead). **Kangcheng Zhang**: Data curation (equal); formal analysis (equal); investigation (equal); methodology (equal); resources (equal); software (equal); validation (equal). **Yunfeng Zhao**: Data curation (equal); investigation (equal); methodology (equal); resources (equal); software (equal). **Yexin Zhao**: Investigation (equal). **Bianbian Wang**: Investigation (equal). **Yi Wang**: Investigation (equal). **Tangqing He**: Methodology (equal). **Xinyu Xu**: Investigation (equal); methodology (equal). **Tongshuo Bai**: Investigation (equal). **Yi Zhang**: Investigation (equal). **Shuijin Hu**: Conceptualization (lead); data curation (equal); formal analysis (equal); project administration (equal); visualization (equal); writing—original draft (equal); writing—review and editing (lead).

## ETHICS STATEMENT

This article does not contain any studies with human participants or animals.

## CONFLICT OF INTERESTS

The authors declare no conflict of interests.

## Supporting information

Supporting information.

## Data Availability

The representative sequences of ITS and 16S rRNA gene amplicons are available in figshare (https://doi.org/10.6084/m9.figshare.23257013). All other relevant data are available upon reasonable request from the corresponding author (Yunpeng Qiu).
