# Supplementary material for: Climate warming suppresses abundant soil fungal taxa and reduces soil carbon efflux in a semi‐arid grassland
Source: mLife. 2023 Dec 29;2(4):389–400. doi: 10.1002/mlf2.12098 (PMC10989086; doi:10.1002/mlf2.12098)
Supplement: Supplementary file 1 — Supporting information. [file MLF2-2-389-s001.docx]

**Climate warming suppresses soil abundant fungal taxa and reduces soil carbon efflux in a semi-arid grassland**

Yunpeng Qiu^#*^, Kangcheng Zhang^#^, Yunfeng Zhao^#^, Yexin Zhao, Bianbian Wang, Yi Wang, Tangqing He, Xinyu Xu, Tongshuo Bai, Yi Zhang, Shuijin Hu

^#^ These authors contributed equally to this work

*Corresponding author:

Yunpeng Qiu: [yunpeng_qiu@njau.edu.cn](mailto:yunpeng_qiu@njau.edu.cn); phone: +8618852052826


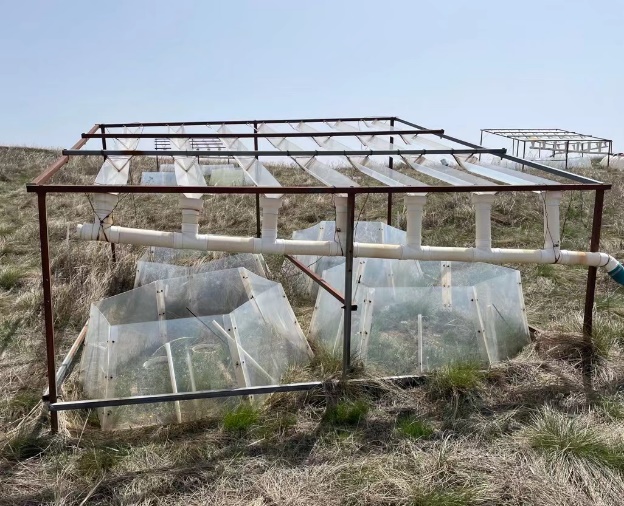


**Figure S1.** A partial overview of field warming and precipitation reduction plots. Warming was simulated via open-top chambers (OTCs). Seven tilted v-shaped transparent plexiglass were placed 1 m high above soil surface to intercept 30% of rainfall to simulate a light to moderate precipitation reduction. Photo credit: Kangcheng Zhang.


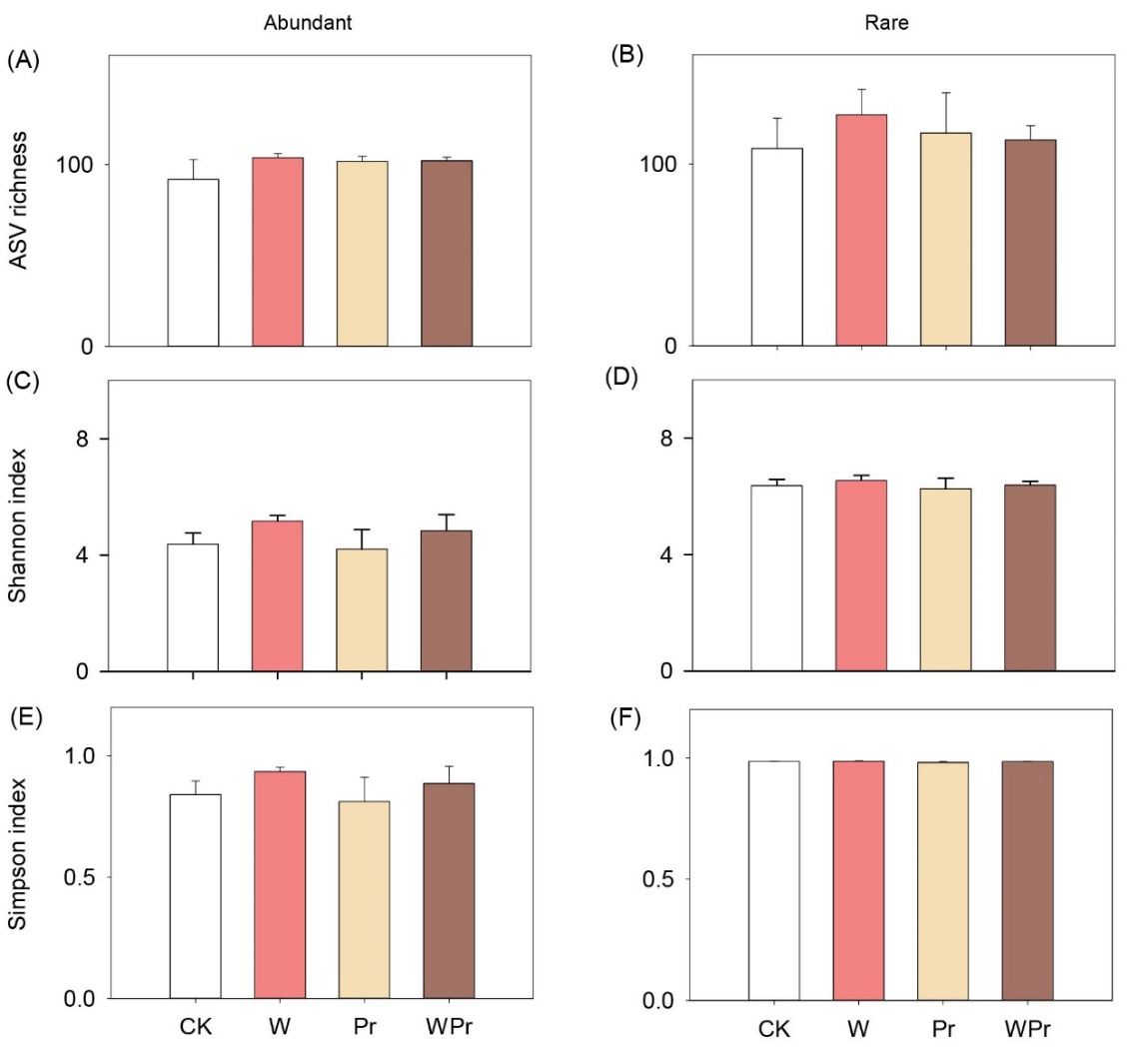


**Figure S2.** Soil fungal alpha diversity. Effects of warming and precipitation reduction on abundant (A, C, E) and rare (B, D, F) fungal alpha diversity. Values are means ± SE (n = 4). The four treatments are as follows: CK, control; W, warming; Pr, precipitation reduction; WPr, combination of warming and precipitation reduction.

**
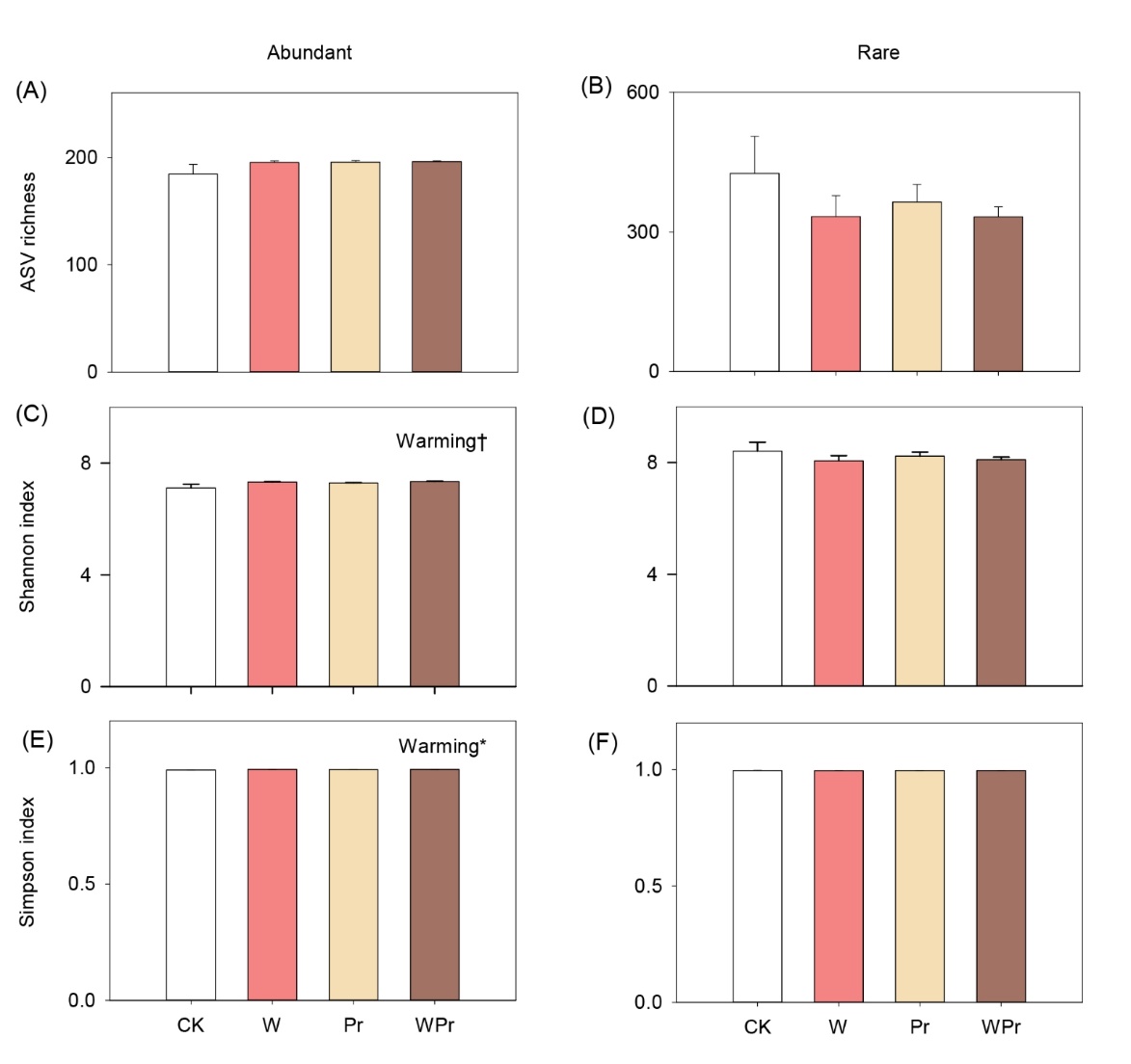
**

**Figure S3.** Soil bacterial alpha diversity. Effects of warming and precipitation reduction on abundant (A, C, E) and rare (B, D, F) bacterial alpha diversity. Values are means ± SE (n = 4). The four treatments are as follows: CK, control; W, warming; Pr, precipitation reduction; WPr, combination of warming and precipitation reduction. The statistically significant effects of warming, precipitation reduction (PR) and their interaction (Warming×PR) are indicated: ^†^0.05 < *p* ≤ 0.10; *0.01 < *p* ≤ 0.05, ANOVA mixed model.


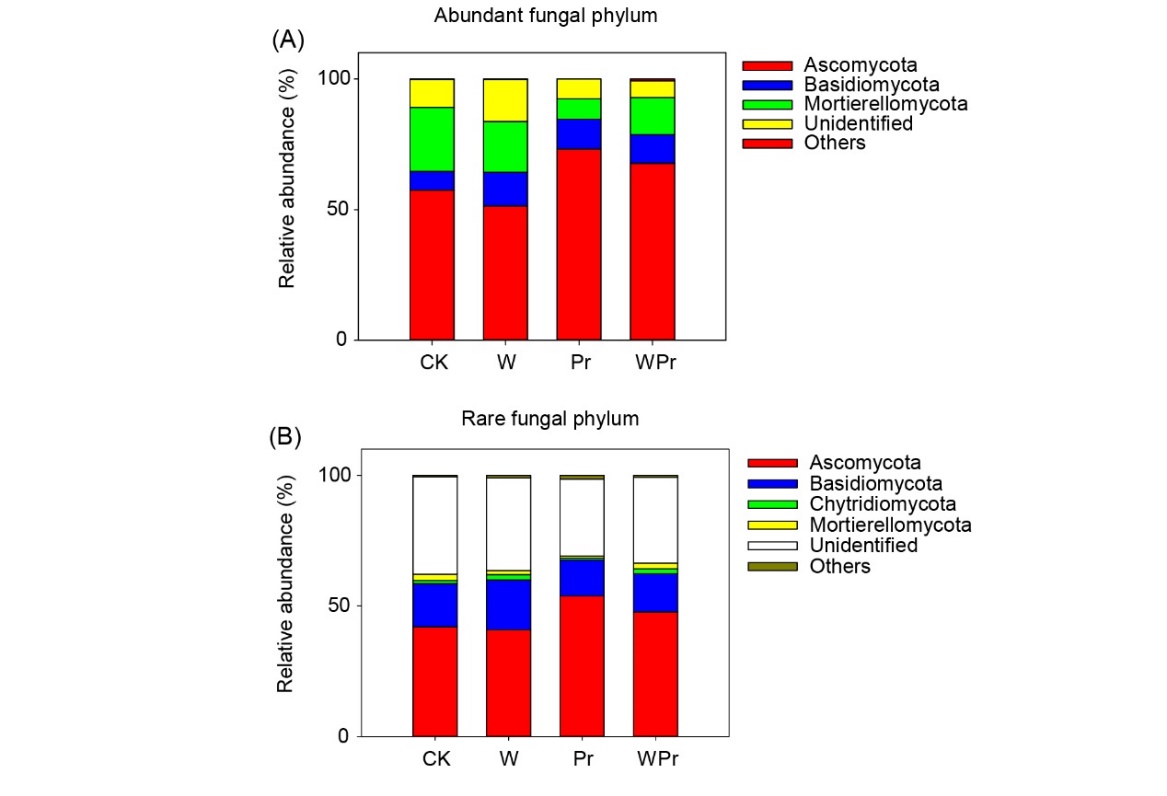


**Figure S4.** Soil fungal community composition at phylum level. Effects of warming and precipitation reduction on the composition of abundant (A) and rare (B) fungal taxa at phylum level. The four treatments are as follows: CK, control; W, warming; Pr, precipitation reduction; WPr, combination of warming and precipitation reduction.

**
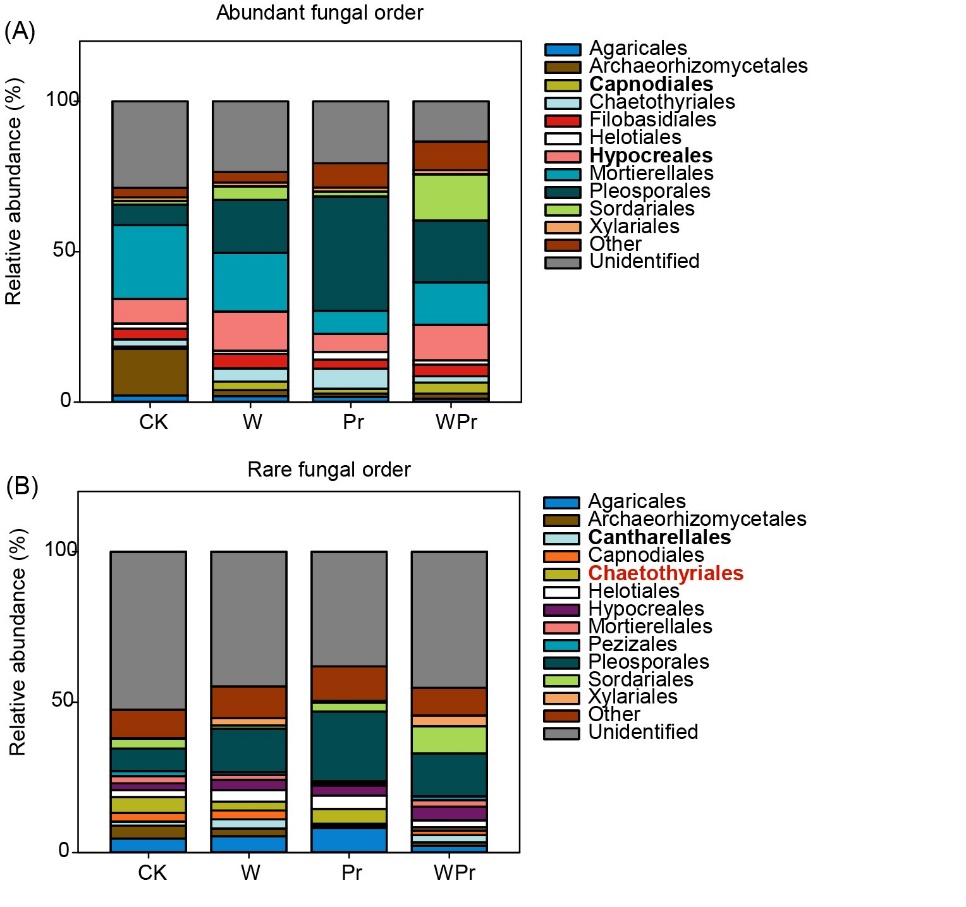
**

**Figure S5.** Soil fungal community composition at order level**.** Effects of warming and precipitation reduction on the composition of abundant (A) and rare (B) fungal taxa at order level. Black bold denotes positive effect of warming on the order and the red bold denotes negative effect of warming on the order. The four treatments are as follows: CK, control; W, warming; Pr, precipitation reduction; WPr, combination of warming and precipitation reduction.


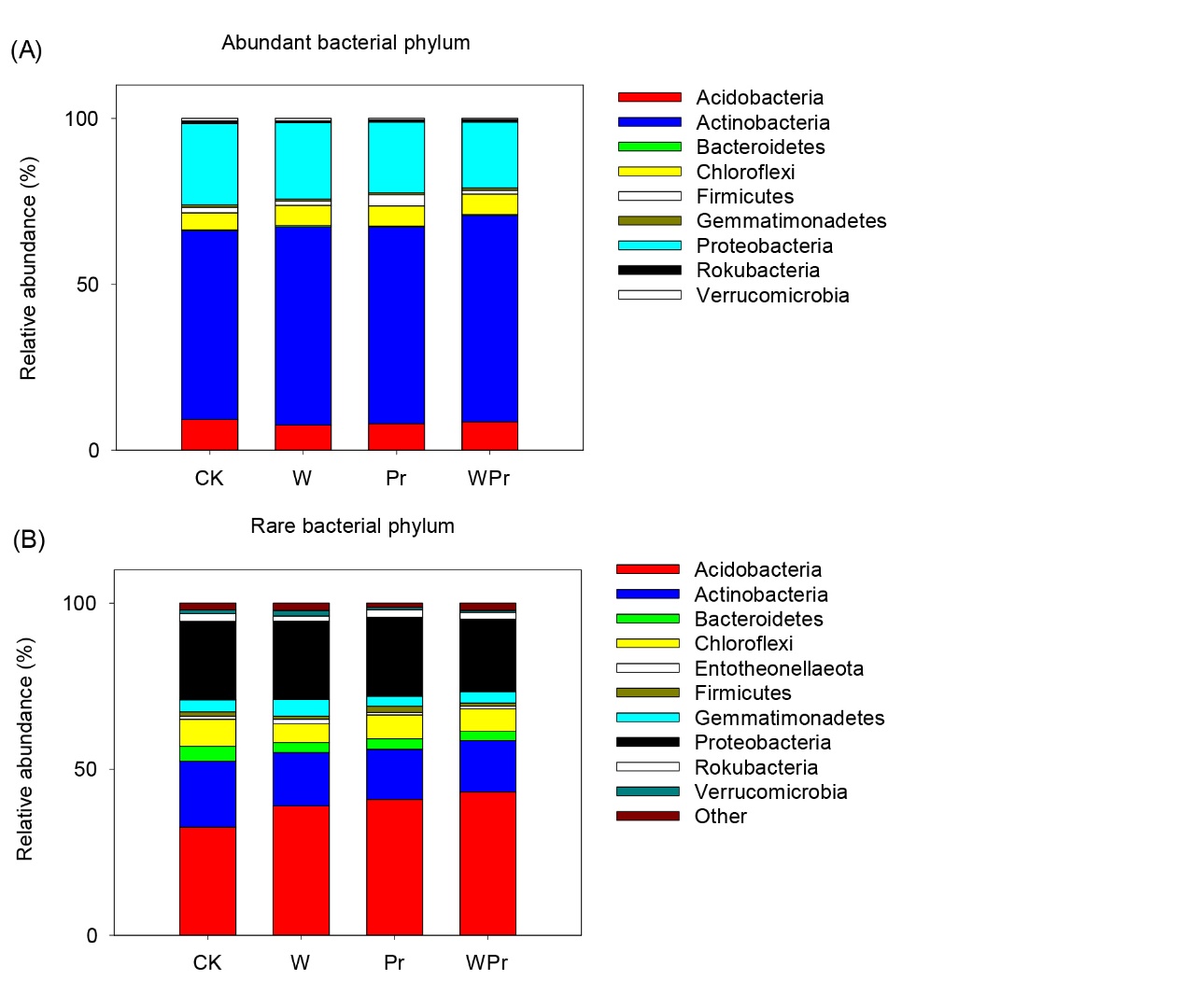


**Figure S6.** Soil bacterial community composition at phylum level. Effects of warming and precipitation reduction on the composition of abundant (A) and rare (B) bacterial taxa at phylum level. The four treatments are as follows: CK, control; W, warming; Pr, precipitation reduction; WPr, combination of warming and precipitation reduction.


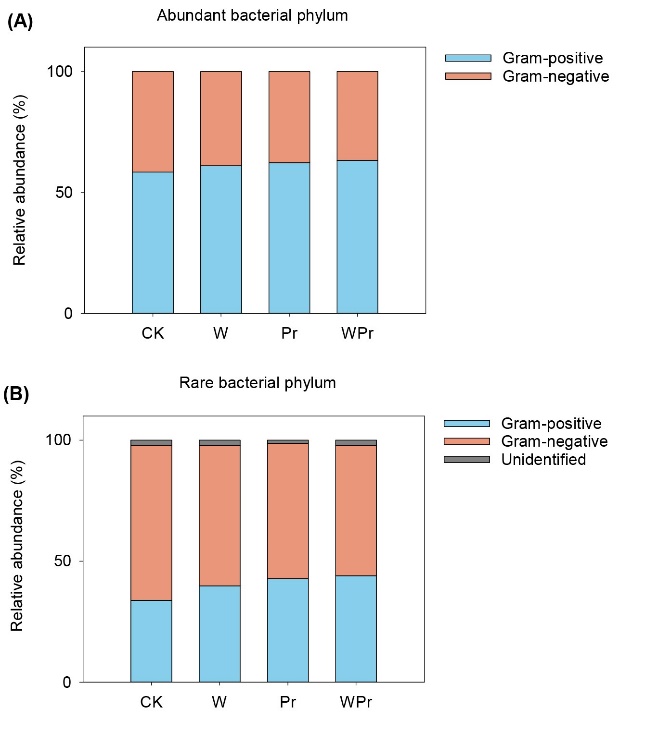


**Figure S7. Gram-positive and gram-negative bacteria.** Effects of warming and precipitation reduction on gram-positive and negative bacterial abundance inferred from the sequencing data. The four treatments are as follows: CK, control; W, warming; Pr, precipitation reduction; WPr, combination of warming and precipitation reduction.

**
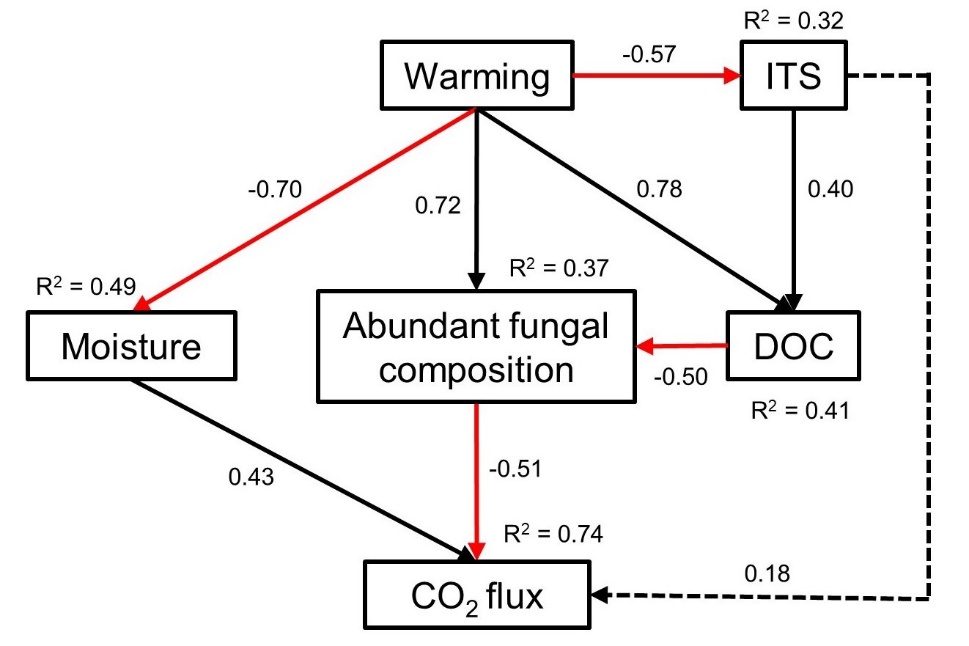
**

**Figure S8.** Potential drivers of soil CO_2_ flux. Structural equation modelling (SEM) analysis of the effects of warming on soil CO_2_ emissions. Results of the final model ﬁtting: Chi-square (χ^2^) = 6.658, *P* = 0.354, degree of freedom (df) = 6, comparative ﬁt index (CFI) = 0.986, root square mean error of approximation (RMSEA) = 0.083. Abundant fungal community composition is indicated by the first axis of PCoA. ITS, the abundance of soil fungi; DOC, dissolved organic carbon. Values associated with arrows are standardized path coefficients. Black arrows indicate significant positive relationships and red arrows indicate significant negative relationships (*P* < 0.05). The dashed arrows indicate non-significant relationship (*P* > 0.05). *R^2^* values are the proportion of variation explained by relationships with other variables.

**Table S1.** Primers and conditions used for real-time quantitative PCR of fungal ITS and genes.

| **Primer set** | **Target**  **genes** | **Amplicon**  **length (bp)** | **Thermal profile** | **References** |
| --- | --- | --- | --- | --- |
| ITS5  (GGAAGTAAAAGTCGTAACAAGG)  ITS2  (GCTGCGTTCTTCATCGATGC) | ITS | 280 | 95°C, 3min; 40 cycles (95°C, 30s; 55°C, 30s; 72°C, 45s) | Caporaso et al. 2011 |
| 338F  (ACTCCTACGGGAGGCAGCA)  806R  (GGACTACHVGGGTWTCTAAT) | 16S rRNA | 500 | 95°C, 3min; 40 cycles (95°C, 30s; 60°C, 30s; 72°C, 45s) | Lee et al. 2012 |

Caporaso JG, Lauber CL, Walters WA, Berg-Lyons D, Lozupone CA, Turnbaugh PJ, et al. Global patterns of 16S rRNA diversity at a depth of millions of sequences per sample. *Proc Natl Acad Sci USA.* 2011;108:4516–522.

Lee CK, Barbier BA, Bottos EM, McDonald IR, Cary SC. The Inter-Valley Soil Comparative Survey: the ecology of Dry Valley edaphic microbial communities.*ISME J.* 2012;6:1046–1057.

**Table S2.** Results (*P* values) of ANOVA for the effects of warming and precipitation reduction (PR) on the soil bacterial, fungal abundance, and the ratio of fungal to bacterial abundance, and mean soil CO_2_ flux.

| Variables | Warming | PR | Warming×PR |
| --- | --- | --- | --- |
| 16S rRNA | 0.735 | 0.975 | 0.230 |
| ITS | **0.009** | 0.576 | **0.027** |
| ITS/16S rRNA | **0.019** | 0.671 | 0.377 |
| Mean CO_2_ flux | **0.020** | 0.485 | 0.824 |

**Table S3.** Results (*P* values) of ANOVA for the effects of warming and precipitation reduction (PR) on the relative abundance of abundant and rare fungal and bacterial taxa.

|  | Variables | Warming | PR | Warming×PR |
| --- | --- | --- | --- | --- |
| Fungi | Abundant | 0.237 | 0.266 | 0.789 |
|  | Rare | 0.544 | 0.335 | 0.732 |
| Bacteria | Abundant | 0.138 | 0.970 | 0.307 |
|  | Rare | 0.124 | 0.275 | 0.247 |

**Table S4.** Results (*P* values) of ANOVA for the effects of warming and precipitation reduction (PR) on alpha diversities of abundant and rare fungi.

|  | Variables | Warming | PR | Warming×PR |
| --- | --- | --- | --- | --- |
| Abundant | Richness | 0.320 | 0.559 | 0.338 |
|  | Shannon | 0.151 | 0.604 | 0.884 |
|  | Simpson | 0.194 | 0.534 | 0.877 |
| Rare | Richness | 0.623 | 0.943 | 0.329 |
|  | Shannon | 0.521 | 0.986 | 0.371 |
|  | Simpson | 0.452 | 0.890 | 0.384 |

**Table S5.** Results (*P* values) of ANOVA for the effects of warming and precipitation reduction (PR) on alpha diversities of abundant and rare bacteria.

|  | Variables | Warming | PR | Warming×PR |
| --- | --- | --- | --- | --- |
| Abundant | Richness | 0.244 | 0.226 | 0.286 |
|  | Simpson | **0.032** | 0.166 | 0.324 |
|  | Shannon | *0.080* | 0.180 | 0.272 |
| Rare | Richness | 0.230 | 0.539 | 0.546 |
|  | Simpson | 0.243 | 0.911 | 0.596 |
|  | Shannon | 0.236 | 0.687 | 0.568 |

**Table S6.** Results (*P* values) of ANOVA for the effects of warming and precipitation reduction (PR) on soil abundant (i.e., *Archaeorhizomycetales****,*** *Capnodiales* and *Hypocreales*) and rare fungal order (i.e., *Cantharellales* and *Chaetothyriales*).

|  | Variables | Warming | PR | Warming × PR |
| --- | --- | --- | --- | --- |
| Abundant | *Archaeorhizomycetales* | 0.246 | 0.189 | 0.203 |
|  | *Capnodiales* | **0.003** | 0.182 | 0.957 |
|  | *Hypocreales* | **0.046** | 0.479 | 0.842 |
|  | *Capnodiales /* *Archaeorhizomycetales* | 0.291 | 0.212 | 0.558 |
| Rare | *Cantharellales* | **0.010** | 0.184 | 0.830 |
|  | *Chaetothyriales* | **0.044** | 0.442 | 0.610 |

**Table S7.** Correlation coefficients (Pearson) between soil CO_2_ efflux, soil properties and the abundance of ITS and 16S rRNA genes across experiment treatments.

|  | NH_4_^+^ | NO_3_^-^ | DOC | MBC | MBN | NMR | CO_2_ | ITS | 16S rRNA |
| --- | --- | --- | --- | --- | --- | --- | --- | --- | --- |
| NH_4_^+^ | 1.000 | -.030 | -.003 | *.479* | .401 | .237 | **.579*** | **.617*** | .370 |
| NO_3_^-^ | -.030 | 1.000 | -.138 | .122 | -.227 | .362 | -.032 | -.003 | .095 |
| DOC | -.003 | -.138 | 1.000 | *-.433* | -.066 | **-.520** | -.092 | -.041 | .253 |
| MBC | *.479* | .122 | *-.433* | 1.000 | .001 | .356 | *.475* | *.469* | .146 |
| MBN | .401 | -.227 | -.066 | .001 | 1.0000 | .044 | .259 | -.007 | .245 |
| NMR | .237 | .362 | **-.520** | .356 | .044 | 1.0000 | .421 | .308 | .091 |
| CO_2_ | **.579*** | -.032 | -.092 | *.475* | .259 | .421 | 1.000 | **.698**** | .385 |
| ITS | **.617*** | -.003 | -.041 | *.469* | -.007 | .308 | **.698**** | 1.000 | .409 |
| 16S rRNA | .370 | .095 | .253 | .146 | .245 | .091 | .385 | .409 | 1.000 |

DOC, dissolved organic carbon; MBN, soil microbial biomass nitrogen; MBC, soil microbial biomass carbon; NMR, net nitrogen mineralization rates. Bold values indicate a significant different at *P* < 0.05.

**Table S8.** Mantel test (Bray-Curtis) results for the relationships between fungal community composition (total, abundant and rare) and soil properties or soil CO_2_ efflux.

| Variables | Total | |  | Abundant | |  | Rare | |
| --- | --- | --- | --- | --- | --- | --- | --- | --- |
|  | *r value* | *P value* |  | *r value* | *P value* |  | *r value* | *P value* |
| NH_4_^+^-N | 0.312 | **0.049** |  | 0.331 | **0.034** |  | 0.016 | 0.425 |
| NO_3_^−^-N | 0.308 | 0.076 |  | 0.299 | 0.082 |  | 0.311 | **0.047** |
| DIN | 0.368 | **0.045** |  | 0.358 | **0.049** |  | 0.329 | **0.043** |
| DOC | -0.003 | 0.477 |  | -0.010 | 0.486 |  | -0.047 | 0.623 |
| MBC | 0.225 | 0.131 |  | 0.240 | 0.114 |  | 0.125 | 0.170 |
| MBN | -0.038 | 0.555 |  | -0.033 | 0.540 |  | -0.063 | 0.680 |
| NMR | 0.113 | 0.275 |  | 0.117 | 0.258 |  | -0.045 | 0.592 |
| CO_2_ efflux | 0.397 | **0.035** |  | 0.419 | **0.027** |  | 0.096 | 0.254 |

DIN, dissolved inorganic nitrogen; DOC, dissolved organic carbon; NMR, net nitrogen mineralization rates; MBN, soil microbial biomass nitrogen; MBC, soil microbial biomass carbon. Bold values indicate a significant different at *P* < 0.05.
